# Thoracolumbar Interfascial Plane (TLIP) block verses other paraspinal fascial plane blocks and local infiltration for enhanced pain control after spine surgery: a systematic review

**DOI:** 10.1186/s12871-024-02500-1

**Published:** 2024-03-28

**Authors:** Tarika D. Patel, Meagan N. McNicholas, Peyton A. Paschell, Paul M. Arnold, Cheng-ting Lee

**Affiliations:** 1 Carle Illinois College of Medicine, Champaign, IL USA; 2 Carle Neuroscience Institute, Carle Foundation Hospital, Urbana, IL USA; 3 Department of Anesthesiology, Carle Foundation Hospital Urbana, Illinois, USA

**Keywords:** Thoracolumbar interfascial plane block, Spine surgery, Pain management, Analgesia, Systematic review

## Abstract

Spinal surgeries are accompanied by excessive pain due to extensive dissection and muscle retraction during the procedure. Thoracolumbar interfascial plane (TLIP) blocks for spinal surgeries are a recent addition to regional anesthesia to improve postoperative pain management. When performing a classical TLIP (cTLIP) block, anesthetics are injected between the muscle (m.) multifidus and m. longissimus. During a modified TLIP (mTLIP) block, anesthetics are injected between the m. longissimus and m. iliocostalis instead. Our systematic review provides a comprehensive evaluation of the effectiveness of TLIP blocks in improving postoperative outcomes in spinal surgery through an analysis of randomized controlled trials (RCTs).

We conducted a systematic review based on the PRISMA guidelines using PubMed and Scopus databases. Inclusion criteria required studies to be RCTs in English that used TLIP blocks during spinal surgery and report both outcome measures. Outcome data includes postoperative opioid consumption and pain.

A total of 17 RCTs were included. The use of a TLIP block significantly decreases postoperative opioid use and pain compared to using general anesthesia (GA) plus 0.9% saline with no increase in complications. There were mixed outcomes when compared against wound infiltration with local anesthesia. When compared with erector spinae plane blocks (ESPB), TLIP blocks often decreased analgesic use, however, this did not always translate to decreased pain. The cTLIP and mTLP block methods had comparable postoperative outcomes but the mTLIP block had a significantly higher percentage of one-time block success.

The accumulation of the current literature demonstrates that TLIP blocks are superior to non-block procedures in terms of analgesia requirements and reported pain throughout the hospitalization in patients who underwent spinal surgery. The various levels of success seen with wound infiltration and ESPB could be due to the nature of the different spinal procedures. For example, studies that saw superiority with TLIP blocks included fusion surgeries which is a more invasive procedure resulting in increased postoperative pain compared to discectomies.

The results of our systematic review include moderate-quality evidence that show TLIP blocks provide effective pain control after spinal surgery. Although, the application of mTLIP blocks is more successful, more studies are needed to confirm that superiority of mTLIP over cTLIP blocks. Additionally, further high-quality research is needed to verify the potential benefit of TLIP blocks as a common practice for spinal surgeries.

## Introduction

Spinal surgeries are often accompanied by excessive pain due to extensive tissue dissection and muscle retraction during the procedure [1, 2]. Effective pain control is a crucial aspect of patient comfort and a pivotal determinant of overall surgical outcomes. Regional anesthesia techniques have gained prominence in the quest for optimal analgesia, with thoracolumbar interfascial plane (TLIP) blocks emerging as a noteworthy option.

Opioids are commonly used for post-spinal surgery pain management [3, 4]. While opioids provide effective analgesia, their use is associated with reoperations and can lead to undesired outcomes such as long-term dependence, nausea, vomiting, and respiratory depression. Multimodal analgesic regimens are the use of 2 or more analgesics or techniques to reduce the dose of each individual drug and can help with the goal of reducing opioid use while providing adequate pain control [5, 6]. While there is no one optimal analgesic combination, unless there are patient-specific contraindications, all patients should receive a combination of acetaminophen and nonsteroidal anti-inflammatory drugs (NSAIDs) perioperatively or intraoperatively and continued postoperatively as scheduled dosing. Furthermore, all patients should receive surgical site infiltration and/or regional anesthesia (interfascial plane or peripheral nerve block). While opioid use should be reduced, the role of opioid-free analgesia remains controversial. In the acute postoperative period, opioids should be administered only as a rescue agent. Intravenous (IV) analgesic should be limited with the goal of transferring patients to oral medications and not impede ambulation and rehabilitation [7]. Therefore, a multimodal pain regimen is key to improving patient outcomes and reducing total opioid consumption.

In 2015, Hand et al. [8] introduced the classical TLIP (cTLIP) block, which targets the dorsal rami of the thoracolumbar nerves.. This is a relatively recent addition to regional anesthesia techniques for spinal surgeries. It involves the precise administration of local anesthetics between the multifidus and longissimus paraspinal muscles at the third lumbar vertebra often assisted by ultrasound. It is often difficult to delineate between the two muscles, however, lumbar extension can help improve the visualization of the intended injection site. This technique is designed to selectively target the sensory innervation of the thoracolumbar region, potentially offering a valuable alternative to systemic opioids. To improve upon the challenges and difficulties seen with the cTLIP block, Ahiskalioglu et al. [9], in 2017, introduced the modified TLIP (mTLIP) block where anesthetics are instead injected between the longissimus and iliocostalis muscles. The erector spinae plane block (ESPB) is similar to the TLIP block, however, an ESPB targets both the ventral and dorsal rami of the thoracic and abdominal spinal nerves by injecting anesthetics between the erector spinae muscle and transverse processes of vertebrae. By targeting only the dorsal rami of spinal nerves, the TLIP block provides more focused dermatomal coverage for back muscles which could lead to better controlled postoperative pain [10]. 

Our systematic review endeavors to provide a comprehensive evaluation of the effectiveness of TLIP blocks in improving postoperative outcomes in spinal surgery. The primary objectives encompass a multifaceted exploration of the impact of TLIP blocks for patients undergoing lumbar spinal surgery, focusing on postoperative pain control, opioid consumption, and the incidence of complications. We aim to provide a nuanced understanding of how TLIP blocks fare in comparison to other anesthesia modalities commonly employed in spinal surgery through a meticulous analysis of randomized controlled trials.

## Methods

We conducted a systematic review based on the Preferred Reporting items for Systematic Reviews and Meta-Analyses (PRISMA) guidelines. PubMed, Scopus, and clinicaltrials.gov were the databases used. The search strategy was focused on “thoracolumbar interfascial plane blocks” for “spine” surgeries. Multiple search phrases and keywords were used to limit bias and capture missed studies that may not have shown up using a single search. The snowball method was used to collect references from other systematic reviews for potentially relevant articles that were missed with the initial search. At the start, all abstracts were read in their entirety for initial screening. The full text of studies with potential for final inclusion were evaluated for eligibility based on inclusion and exclusion criteria. Each article was reviewed by two independent researchers to determine inclusion based on our pre-determined criteria, then was confirmed by a third reviewer.

Inclusion criteria required studies to be randomized control trials (RCTs) in the English language that evaluated the impact of TLIP blocks during spinal surgery on postoperative pain and analgesia. Cohort studies were not included because most were redacted, and case studies provided minimal quantifiable outcome measurements. Inclusion criteria included the use of TLIP blocks for any type of spine-based surgery and report standardized outcome measures of both postoperative analgesic use and pain. Studies that are not randomized control trials of human patients, do not report any outcome data, and involve surgery beyond the spine were excluded.

We collected data regarding age range, total number of participants, type of surgery, treatment characteristics, and type of anesthesia mixture. Outcome data include intraoperative and postoperative opioid consumption, time to postoperative analgesia, and postoperative pain. Complications were also collected for each study. Continuous variable data was reported as a mean ± standard deviations or a median (interquartile range). Categorical variable data were reported as frequency with percentages. Associations were reported with statistical significance at a *p*-value < 0.5. Studies were grouped based on the type of control TLIP blocks were compared against.

The critical appraisal of included studies was evaluated using the JBI assessment tool for risk of bias for randomized controlled trials [11]. This tool includes 11 items that aim to assess for a variety of biases such as selection, performance, and measurement. Each question can receive an answer of yes, no, unclear, or not applicable. Studies with a higher number of answers to be yes have a low risk of bias and those with a higher number of answers to be no have a high risk of bias.

## Results

**Fig. 1:**
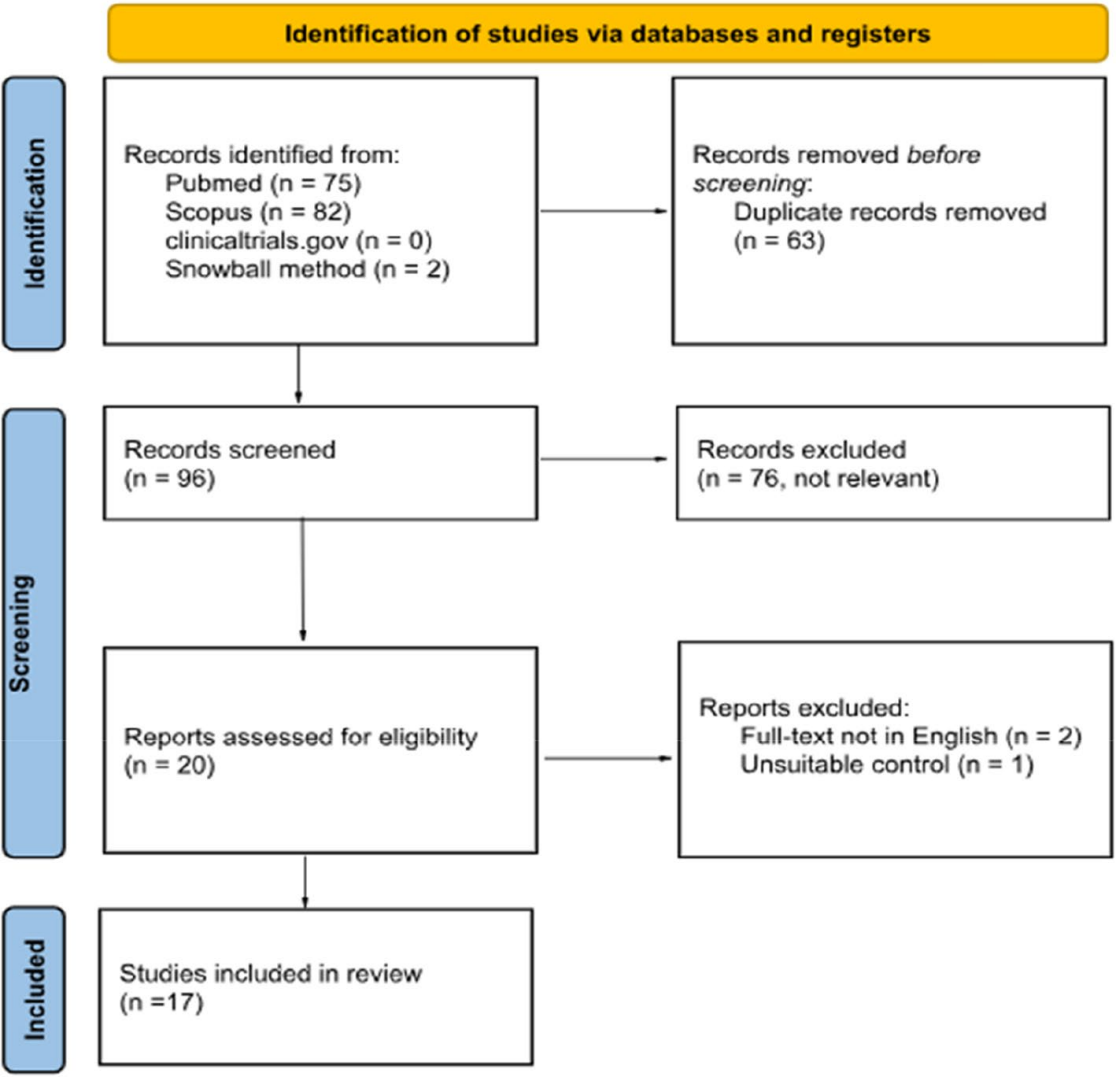
PRISMA flow diagram for study selection

A total of 17 RCTs were included in this study with the age of patients ranging from 18–74 years (Fig. 1). Risk of bias was moderate to high given there were several different areas where there were doubts if criteria were met for each study (Table 1). Only two studies, Chen et al. [12] and Ahiskalioglu et al., [13] met all criteria, leading to a low risk of bias. Additionally, only three studies met the criteria where those delivering the treatment were blind to treatment assignment (question 5).

**Table 1:**
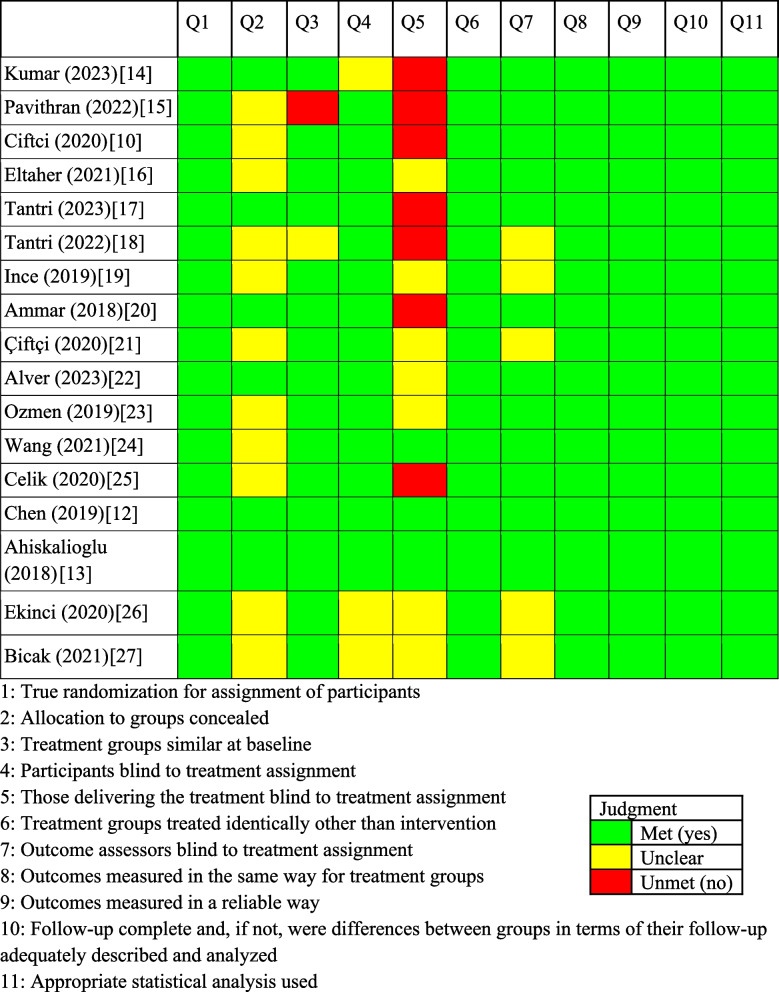
Risk of bias analysis on included studies [14, 15, 10, 16–25, 12, 13, 26, 27]

The types of surgeries performed, most often at the lumbar level, include discectomies, fusions, and decompression/stabilization procedures. TLIP blocks were performed after induction with general anesthesia (GA) either by the modified or classical method. The TLIP blocks were often compared to either GA plus 0.9% saline (*n* = 5), wound infiltration (*n* = 4), ESPB (*n* = 4), quadratus lumborum block (QLB) (*n* = 1), or epidural analgesia (*n* = 1). Two studies compared the two modes of TLIP blocks, classical and modified (Table 2).

**Table 2:** Main outcome measurements of studies included in this review

Author (year)	Age (years)	Surgery type	Treatment groups	Anesthesia	Pain (VAS)	Analgesia and Opioids	Adverse events
Kumar (2023) [14]	18–60	lumbar discectomy, lumbar laminoplasty	ESPB (*n* = 30) mTLIP (*n* = 30)	20 ml 0.2% ropivacaine bilaterally	**24 h:** 1.5 (1–2.25) vs 3 (2–3.25)^a^ **48 h:** 2 (1–2) vs 2 (2–2)	**48 h fentanyl (ug):** 124.16 ± 80.8 vs 189.7 ± 141.1^a^	No incidences ofLAST in either group
Pavithran (2022) [15]	20–60	posterior lumbar spine fusion	WI (*n* = 35) cTLIP (*n* = 36)	40 ml 0.375% ropivacaine and 10 ml 2% lignocaine; 25 mL bilaterally	**48 h:** 6 (4–7) vs 4 (3–5)^a^	**Total tramadol (mg):** 220.3 ± 86.9 vs 100 ± 40.8^a^ **% requiring rescue analgesia:** 91.4 vs 27.8 **Time to first analgesia (min):** 340 (180–360) vs 1440 (1290–2280)^a^	
Ciftci (2020) [10]	18–65	lumbar discectomy	GA (*n* = 30) ESPB (*n* = 30) mTLIP (*n* = 30)	20 mL of 0.25% BPVC bilaterally	**24 h:** 0 (0–1) vs 0 (0–1) vs 0 (0–1)	**Total opioids (mcg):** 140 (80–260)^a^ ^ vs 20 (0–140)^a^ vs 20 (0–140)^ **% requiring rescue analgesia:** 70^a^ ^ vs 30^a^ vs 23^	**% Nausea:** 43^a^ ^ vs 10^a^ vs 10^ **% Vomiting:** 23 vs 6 vs 10 **% Itching:** 13 vs 23 vs 13
Eltaher (2021) [16]	> 21	Elective spine surgery	GA + NS (*n* = 30) cTLIP (*n* = 30)	20 mL 0.25% BPVC bilaterally	**24 h (NRS at rest):** 3.43 ± 0.5 vs 2.53 ± 0.97^a^ **24 h (NRS at passive flexion):** 3.93 ± 0.78 vs 2.73 ± 0.87^a^	**24 h morphine (mg):** 14.33 ± 2.58 vs 5.13 ± 1.55^a^ **Time to first analgesia (hrs):** 0.92 ± 1.23 vs 7.30 ± 2.69^a^	
Tantri (2023) [17]	18–65	posterior lumbar decompression	ESPB (*n* = 20) cTLIP (*n* = 20)	20 mL 0.25% BPVC bilaterally	**24 h (NRS):** 3 vs 3.5	**24 h morphine (mg):** 10 (5) vs 7 (3) **Time to first analgesia (min):** 300 (154) vs 548 (319)^a^	
Tantri (2022) [18]	18–65	posterior lumbar stabilization	cTLIP (*n* = 4) mTLIP (*n* = 4)	20 mL 0.25% of BPVC bilaterally	**6 h (NRS):** 3.75 ± 1.7 vs 2.75 ± 1.5 **12 h (NRS):** 4 ± 1.6 vs 3.5 ± 1.3	**24 h morphine (mg):** 3.5 ± 1.3 vs 3.25 ± 1.3	
Ince (2019) [19]	18–85	single level discectomy	WI (*n* = 20) TLIP (*n* = 20)	20 mL mixture of BPVC and lidocaine bilaterally	**24 h (NRS):** 2.70 ± 1.1 vs 2.60 ± 1.1	**24 h total fentanyl:** 282.0 ± 226.63 vs 206.0 ± 163.0	
Ammar (2018) [20]	21–60	single-level or multiple level lumbar discectomy	GA + NS (*n* = 35) cTLIP (*n* = 35)	20 ml mixture of 0.25% BPVC and 1% lidocaine bilaterally	**24 h (at rest):** 4 (4–4) vs 3.5 (3–4)^a^ **24 h (movement):** 6 (5–6) vs 5 (4–6)	**24 h total morphine (mg):** 25.88 ± 5.17 vs 9.7 ± 6.38^a^ **Time to first analgesia (min):** 82 ± 69.01 vs 442.7 ± 126.47^a^	**Sedation score:** 3 (2–4) vs 2 (2–2)^a^ **Nausea score:** 0 (0–1) vs 0 (0–0)
Çiftçi (2020) [21]	18–65	single-level lumbar discectomy	cTLIP (*n* = 30) mTLIP (*n* = 30)	20 mL 0.25% BPVC bilaterally	**24 h (passive):** 0.13 ± 0.34 vs 0.37 ± 0.49^a^ **24 h (active):** 0.5 ± 0.68 vs 0.47 ± 0.57	**0–8 h fentanyl:** 22.66 ± 27.65 vs 29.33 ± 25.04 **8–16 h fentanyl:** 5.33 ± 13.82 vs 6 ± 11.91 **16–24 h fentanyl:** 3.33 ± 9.22 vs 2.66 ± 6.91	**% Nausea:** 6.6 vs 10 **% Vomiting:** 6.6 vs 10 **% Itching:** 23.3 vs 13.3
Alver (2023) [22]	18–65	microscopic discectomy	QLB (*n* = 30) mTLIP (*n* = 30)	30 mL 0.25% bupivicaine bilaterally	**16 h (static NRS):** 0.93 ± 0.58 vs 0.33 ± 0.71^a^ **16 h (dynamic NRS):** 1.07 ± 0.79 vs 0.43 ± 0.77^a^	**% Need of rescue analgesia**: 26.6 vs 23.3	**% Nausea:** 23.3 vs 20 **% Vomiting:** 20 vs 16.6 **% Itching:** 13.3 vs 10
Ozmen (2019) [23]	18–60	Single level herniated lumbar disk surgery	GA + NS (*n* = 40) mTLIP (*n* = 40)	20 mL bupivicaine bilaterally	Mean static NRS pain scores were lower in the mTLIP block group, while mean dynamic pain scores were similar	**Total fentanyl (mcg):** 742.5 ± 220.3 vs 446.0 ± 241.98^a^	**% Nausea:** 35 vs 20
Wang (2021) [24]	No age range given	lumbar spine fusion	GA (*n* = 102) ESPB (*n* = 100) TLIP (*n* = 102)	30 mL 0.375% ropivicaine injected bilaterally	Mean static and dynamic pain scores were significantly in favor of mTLIP block group	**Perioperative sufentanil (ug):** 42.85 ± 7.84^a^ ^ vs 28.72 ± 3.99^a^ vs 27.82 ± 3.88^ **Frequency PCA compressions:** 4.78 ± 0.6 vs 2.69 ± 0.8^a^ vs 3.28 ± 0.78^a^	**% Nausea/vomiting:** 20.6 vs 14.7 vs 14 **% Itching:** 10 vs 3.9 vs 6
Çelik (2020) [25]	18–70	single-level lumbar discectomy	Epidural (*n* = 30) mTLIP (*n* = 30)	20 mL 0.25% bupivicaine bilaterally	**24 h:** 4.43 ± 0.72 vs 3.60 ± 0.93^a^	**Total tramadol (mg):** 434.66 ± 86.61 vs 351.33 ± 46.29^a^ **% rescue analgesia:** 36.6 vs 10^a^	Not significant between groups
Chen (2019) [12]	21–74	lumbar spinal fusion surgery	GA + NS (*n* = 30) cTLIP (*n* = 30)	30 ml of 0.375% ropivacaine bilaterally	Pain VAS scores were significantly lower with TLIP block group for up to 36 h	**Total sufentanil (ug):** 42.50 ± 6.40 vs 26.67 ± 5.31^a^ **Frequency of PCA compressions:** 6.47 ± 0.90 vs 3.87 ± 0.94^a^	**% Nausea/vomiting:** 10 vs 8.3 **% Itching:** 5 vs 5
Ahiskalioglu (2018) [13]	18–65	two- or three-level posterior lumbar surgery	GA + NS (*n* = 20) mTLIP (*n* = 20)	20 mL 0.25% bupivacaine bilaterally	Pain VAS scores were significantly lower with mTLIP	**24 h fentanyl (ug):** 496 ± 231 vs 289 ± 154^a^	**% Nausea:** 30 vs 3.3^a^
Ekinci (2020) [26]	18–65	lumbar disc surgery	WI (*n* = 30) mTLIP (*n* = 30)	20 mL 0.25% bupivacaine bilaterally	Pain VAS scores were significantly lower with mTLIP for up to 8 h	**24 h fentanyl:** 223.3 ± 74.1 vs 58.0 ± 32.0^a^ **% rescue analgesia:** 50 vs 23^a^	**% Nausea:** 27 vs 7^a^ **% Vomiting:** 17 vs 0^a^ **% Itching:** 20 vs 3^a^
Bicak (2021) [27]	18–75	lumbar disc surgery	WI (*n* = 21) mTLIP (*n* = 21)	20 mL 0.25% bupivacaine bilaterally	**24 h:** 0.74 vs 1.08	**Opioid consumption (mg/kg):** 27.38 ± 44.65 vs 19.04 ± 40.23	**Complication rate (%):** 4.8 vs 4.8

When 2 or more sets of results are reported with “vs,” the scores are reported as control vs treatment; if multiple patients were reported, treatments results were listed in patient numerical order. Data reported as a mean ± standard deviations or a median (interquartile range)

*cTLIF* classical thoracolumbar interfascial plane, *ESPB* erector spinae plane block, *GA* general anesthesia, *h* hours, *kg* kilogram, *LAST* local anesthetic system toxicity, *min* minute, *mg* milligram, *mL* milliliter, *mTLIF* modified thoracolumbar interfascial plane, *NRS* numeric rating scale, *NS* normal saline, *PCA* patient-controlled analgesia, *ug* microgram, *QLB* quadratus lumborum block, *VAS* visual analog scale, *WI* wound infiltration

^a^or ^ indicates statistical significance between groups

The common make-up of the anesthesia provided was bilateral injections of 20 mL of 0.25% bupivacaine (*n* = 10). Other compositions include bilateral injections of 30 mL 0.25% bupivacaine (*n* = 1), 30 mL of 0.375% ropivacaine (*n* = 2), and 20 mL of 0.2% ropivacaine (*n* = 1) along with a mixture of bupivacaine and lidocaine (*n* = 2) and a mixture of ropivacaine and lignocaine (*n* = 1).

The two main outcomes that were analyzed were postoperative pain and opioid consumption. Pain intensity was reported using the visual analog scale (VAS) or the numeric rating scale (NRS), both of which utilize a scale of 1 to 10. Analgesia consumption includes the amount of opioid use, time to first analgesia, percentage of patients requiring rescue analgesia, and frequency of PCA (patient-controlled analgesia) use. The most frequently reported complications of the anesthesia blocks were nausea and vomiting. The rate and incidence of complications were low and insignificant between treatment groups for most studies. Ahiskalioglu, [13] Ciftci [10] and Ekinci [26] were the only studies that reported a significant decrease in nausea with TLIP block.

Overall, the use of a TLIP block for spinal surgery significantly decreases postoperative opioid use and pain compared to using general anesthesia (GA) plus 0.9% saline only with no increase in complications. The time before analgesia was requested significantly increased for patients who received a TLIP block.

When TLIP blocks were compared against wound infiltration of local anesthesia, two studies, Ince et al. [19] and Bicak et al. [27], found wound infiltration was as effective as a TLIP block for postoperative pain relief. On the other hand, Ekinci et al. [26] and Pavithran et al. [15] found TLIP blocks to be superior.

There appeared to be varying levels of success when TLIP blocks were compared with ESPB. Kumar et al. [14] found patients who were given ESPBs reported significantly decreased total opioid consumption and decreased pain for up to 24 h. However, Ciftci et al. [10] saw no difference in analgesic efficacy between ESPBs and TLIP block groups, but compared to those who did not receive either block, postoperative opioid use was significantly decreased. Similarly, Tantri et al. [17] saw no difference in postoperative pain control between the two block groups. However, TLIP block provided a prolonged duration of analgesia as seen by a significantly increased length of time until first analgesia.

TLIP blocks were also compared against a posterior QLB and epidural analgesia. TLIP provided superior analgesia with quality of recovery score (QoR-40), Kaplan–Meier survival analysis, and postoperative pain control favoring patients who received TLIP blocks.

When the two different methods of TLIP blocks were compared against each other, there was no significant difference in terms of postoperative pain and opioid use. However, Ciftci et al. [21] showed that the mTLIP block method had a significantly higher percentage of success of one-time block at 90% compared to 40% with a cTLIP block.

## Discussion

Conventional spinal surgeries often involve extensive dissection of subcutaneous tissues, bones, and ligaments, resulting in a high degree of postoperative pain and a strikingly high use of opioid analgesics [1, 28]. Long-term consequences of postoperative opioid analgesia surrounding dependence and addiction are well documented and a feared sequela of physicians prescribing these medications. One trial demonstrated opioid overuse in spine surgery, with an increase in postsurgical opioid dependence from 0% to nearly 48% of patients who underwent surgical fusion for degenerative scoliosis in the early 2000s to mid-2010s [28]. Effective pain control is thus an important aspect of postoperative care, supporting the clinical value of our study. The use of TLIP blocks during spine surgery has the possibility of providing better postsurgical pain control with the likelihood of decreasing the incidence of chronic pain. However, current studies only evaluate the effect of TLIP on pain during the first few days after surgery. Thus, further research with longer follow-up is needed to better evaluate its effect on chronic pain over the course of weeks to months after surgery.

The use of regional anesthesia is supported by the Enhanced Recovery After Surgery protocols with the goal of minimizing opioid consumption in patients. One novel technique includes the use of TLIP blocks, first introduced by Hand et al. [8]. TLIP blocks involve targeting the dorsal rami of thoracolumbar nerves as they pass through the paraspinal muscles. The TLIP block is analogous to the transversus abdominis plane (TAP) block for abdominal procedures where the ventral rami of the thoracolumbar nerves are targeted instead. Given the success of TAP blocks in providing analgesia, TLIP blocks were hypothesized to provide a similar benefit for spinal surgeries. The accumulation of the current literature demonstrates that TLIP blocks are superior to non-block procedures in terms of analgesia requirements (total opioid use and time to analgesia) and reported pain throughout the hospitalization in patients who underwent spinal surgery.

Hand et al. [8] developed what is now known as the cTLIP block, where the needle is injected at a 30 degree angle from the skin between the muscle (m.) multifidus and m. longissimus, and is advanced from a lateral to medial direction. Ahiskalioglu et al. [9] modified the TLIP block by injecting anesthetics at a 15 degree angle in a medial to lateral direction, between the m. longissimus and m. iliocostalis. The advantages of the mTLIP block are the elimination of the risk of inadvertent neuraxial injection and the increased success rate of the block as the m. longissimus is more easily discernible from the m. iliocostalis than the m. multifidus. Two studies directly comparing the two methods demonstrate similar postoperative analgesic effects, however, the block success rate was significantly higher with the modified version, supporting the conclusions of Ahiskalioglu et al [9]. However, given the limited reports comparing the two methods of the TLIP block, more RCT studies should be conducted to further validate the mTLIP block and its advantages. It is also important to note a proposal for the nomenclature for paraspinal interfascial plane (PIP) blocks given the new variations to the original TLIP block by Hand et al [8]. There is the complication that the paraspinal muscles of the cervical, thoracic, and lumbar region all have different anatomy, and thus a dorsal ramus block technique is specific to each area [29]. Naming the blocks after the target muscle fascia in PIP blocks could offer more clarity. For example, the TLIP block would include the thoracic multifidus plane (TMP) or lumbar multifidus plane (LMP) blocks while the mTLIP block would include the thoracic longissimus plane (TLP) and lumbar longissimus plane (LLP) blocks. The clinical efficiency of wound infiltration with local anesthetics is  questionable, given the various levels of success seen in studies. A systematic review [30] saw only a few RCTs showing a modest reduction in pain intensity, mainly immediately after the operation, and a minor reduction in opioid use with local anesthetic wound infiltration for lumbar spine surgeries. There were mixed reports among RCTs comparing wound infiltration against TLIP blocks. The varying levels of success may be in part due to the nature of the surgery. Ince et al. [19] and Bicak et al. [27] saw no difference in postoperative analgesics, which may be because discectomies are less invasive than spine fusion surgeries. The studies that saw superiority over wound infiltration included patients who underwent lumbar fusion surgeries, providing further support for the conclusion.

ESPBs are another type of fascial plane block where anesthetics are injected between the erector spinae muscles and thoracic transverse processes, blocking the dorsal and ventral rami of the thoracic and abdominal spinal nerves [31]. A RCT by Avis et al. [32] found that lumbar ESPB combined with the Enhance Recovery After Surgery (ERAS) program did not lead to decreased opioid use than with saline after major spine surgery. Furthermore, quality of life at 3 months between the control and treatment group was similar, further demonstrating the limited benefit of the block. On the other hand, several systematic reviews [33–35] found that ESPBs decreased postoperative pain and opioid consumption for those undergoing spinal surgery. However, much of the evidence is low-quality and is insufficient to support the widespread use of ESPBs for spine surgery. There were mixed results regarding the efficacy of TLIP blocks over ESPBs. All but one report saw a clear decrease in analgesia with TLIP blocks; however, this did not always translate to a decrease in pain intensity or difference in complication rate. The slight benefit of TLIP block may be due to the ability to provide more focused analgesia than ESPBs [36]. While the evidence shows that fascial plane blocks improve outcomes after spine surgery, it is difficult to conclude which block is superior given the limited reports available. The decision to perform one technique over the other may be based on physician and institution preference and expertise [37]. 

It is important to note that while some studies show TLIP blocks having a statistically significant decrease in pain, this change in pain perception does not appear to be clinically significant. A study by Smith et al. [38] looked at determining the magnitude of reduction in pain that is meaningful for patients with acute or chronic pain. A reduction in pain intensity by 10–20% is “minimally important”, by ≥ 30% is “moderately important” and by ≥ 50% is “substantially important” by patients. In our review, the mean difference in VAS/NRS pain scores across studies is rarely greater than one and never greater than two. Thus, pain is only reduced by 10–20% and is not likely to provide patients with a meaningful improvement in pain control. Therefore, the true value of TLIP blocks for spine surgery is likely in the reduction of analgesic and opioid consumption.

Our review includes 17 RCTs and provides updates to the previous systematic reviews that included studies now redacted or removed from publication. Such studies were not included in this report to increase the strength and validity of our findings. In general, our results are consistent with the previous conclusions, with some differences. Both meta-analyses found TLIP blocks to drastically reduce opioid use and provide effective pain control compared to no/sham blocks. However, both Ye et al. [39] and Long et al. [40] do not include any studies comparing TLIP blocks against other types of paraspinal blocks. Second, while the number of reports is limited, they also did not mention studies comparing the modified and classic versions of TLIP. Lastly, while the superiority of TLIP blocks over wound infiltration appeared to be dependent on the type of spinal surgery, Ye et al. found TLIP blocks to be superior overall.

## Limitations

Our review has inevitable limitations. First, there is a lack of homogeneity across studies. The heterogeneity is due to differences in the characteristics of the subjects, anesthetic agents and protocol, postoperative analgesic protocol, and type of surgery. Different spinal surgeries with varying levels of invasiveness make comparison between studies more difficult, as less invasive procedures by nature are expected to result in less postoperative pain than their more invasive counterparts. Additionally, slight variations in the formulation of the anesthetic provided and mode of delivery may have resulted in some differences in effectiveness that we were unable to account for. Furthermore, data on outcome measures was reported in different types of metrics, and some variables like the need for rescue analgesia, QoR-40 score, and Bruggemann comfort scale score were sparse across studies. Lastly, there were limited studies that compared TLIP blocks against wound infiltration and other paraspinal blocks and that compared the two modes of TLIP blocks. Overall, the risk of bias among studies was moderate. Thus, the presence of bias lowers the overall quality and confidence of evidence and conclusion.

## Conclusion

The results of our systematic review provide evidence of the effectiveness of TLIP blocks in improving postoperative pain control. TLIP blocks showed improved outcomes after surgery, including lower pain scores and decreased analgesic requirements compared to patients who received no block and wound infiltration. However, when comparing ESPB and TLIP blocks, it is difficult to ascertain the appropriate choice for a nerve block regarding spinal surgeries. mTLIP blocks appear to be superior to cTLIP blocks, but further research is needed to verify this.

## Data Availability

All data generated or analyzed during this study are included in this published article [and its supplementary information files].
